# Multimorbidity and Sickness Absence/Disability Pension in Patients With Cluster Headache and Matched References: A Swedish Register-Based Study

**DOI:** 10.1212/WNL.0000000000201685

**Published:** 2023-03-07

**Authors:** Caroline Ran, Kristina Alexanderson, Andrea C. Belin, Gino Almondo, Anna Steinberg, Christina Sjöstrand

**Affiliations:** From the Department of Neuroscience (C.R., A.C.B.), Karolinska Institutet, SE-171 77; Department of Clinical Neuroscience (K.A., G.A., A.S., C.S.), Karolinska Institutet, SE-171; Department of Neurology (A.S.), Karolinska University Hospital, SE-171 76; and Department of Neurology (C.S.), Danderyd Hospital, SE-182 88 Stockholm, Sweden.

## Abstract

**Background and Objectives:**

Multimorbidity among patients with cluster headache (CH) is considered to be high, but large studies are lacking. The aims were to explore the occurrence of diagnosis-specific multimorbidity among patients with CH and matched references and possible associations of this with their sickness absence and disability pension.

**Methods:**

We performed a register-based study of patients with CH and matched references, regarding their multimorbidity, sickness absence, and disability pension. Data were obtained from 2 nationwide registers: Statistics Sweden's Longitudinal Integration Database for Health Insurance and Labor Market Studies (LISA) (for sociodemographics in 2009, sickness absence, and disability pension in 2010) and The National Board of Health and Welfare's specialized outpatient and inpatient registers for diagnosis-specific health care in 2001–2010 (for identifying patients with CH and multimorbidity, defined by ICD-10 codes). The prevalence and number of net days of sickness absence and/or disability pension in 2010 were calculated, in general and by multimorbidity. Odds ratios (OR) with 95% confidence intervals (CIs) were calculated for comparison of each diagnostic group with references without the chosen morbidity.

**Results:**

We analyzed 3,240 patients with CH, aged 16–64 years, and living in Sweden in 2010 and 16,200 matched references. A higher proportion of patients with CH had multimorbidity (91.9%) than of references (77.6%), OR 3.263 (95% CI 2.861–3.721), both in general and regarding all analyzed diagnostic groups. Differences were particularly high for diagnoses relating to the nervous (CH 51.8% vs references 15.4%), OR 5.922 (95% CI 5.461–6.422), and musculoskeletal (CH 39.0% vs references 23.7%), OR 2.057 (95% CI 1.900–2.227), systems. Multimorbidity rates were overall higher among women in patients with CH (96.4% vs men 89.6%). Patients with CH had a higher mean number of days of sickness absence and disability pension compared with references, 63.15 vs 34.08 days. Moreover, multimorbidity was associated with a higher mean number of such days in patients with CH, 67.25, as compared with references, 40.69 days.

**Discussion:**

The proportions of multimorbidity were high in both patients with CH and references, however, higher in the patients with CH, who also had higher sickness absence and disability pension levels. In particular, CH patients with multimorbidity and of female sex had high sickness absence and disability pension levels.

Cluster headache (CH) is a trigeminal autonomic headache disorder which manifests as recurrent attacks of excruciating pain behind the eye. Attacks occur at a frequency of 1 attack every other day to 8 attacks per day in active periods, which are often interspaced by attack-free episodes (remission) that can last for weeks up to several years. In cases with remission periods shorter than 3 months per year, the phenotype is classified as chronic CH (CCH).^1^ The pain experienced during an attack is known to be the most severe known to human.^2^ Patients with CH in active phase have been reported to have a higher prevalence of depression and feelings of hopelessness.^3^ There is also an increased risk of suicidal thoughts.^4^ CH has been reported to affect the quality of life of patients in active periods more severely than migraine and comparably with migraine during CH remission periods.^5^ It is not known today if depression and suicidal ideation are comorbidities of CH or a consequence of living with recurrent severe pain. Patients with CH are reported to be smokers to a greater extent,^6^ are reported to have a higher body mass index (BMI), and have been suggested to lead a less healthy lifestyle than the general population.^7^ Studies of comorbidities in patients with CH are few and lack replication. Other diagnoses that have been suggested to occur more frequently in patients with CH are sleep apnea, restless legs, bipolar disorder, dental problems, and deviated septum. Inversely, diabetes, gastrointestinal problems, and cardiovascular diseases seem rarer in patients with CH.^8-10^

We recently reported that patients with CH have more sickness absence and/or disability pension days than matched references from the general population in Sweden, both when measured as prevalence^11^ and number of days with sickness absence and/or disability pension.^12^ Sickness absence was defined as having had such benefits from the Swedish Social Insurance Agency for a sickness absence spell lasting more than 14 days. Disability pension was defined as having been granted disability pension benefits from the same agency because of a long-term reduction of work capacity because of morbidity. In the patient with CH group, women and patients older than 35 years, born abroad, and with lower education had both higher sickness absence and disability pension rates.^11,12^ In line with this, a German survey showed that a larger proportion of patients with CCH received invalidity allowance (i.e., disability pension) than a group of episodic patients with CH.^5^ In addition, a study based on self-reported data from the US CLUSTER HEADACHE SURVEY indicates that CH results in a considerable loss of workdays, work-related disability, and loss of employment.^8^ To date, knowledge is lacking on if, and in that case, how higher sickness absence and disability pension in patients with CH correlate with the presence of other morbidities. Increasing the overall knowledge on morbidity and work capacity of patients with CH is of great importance to get better knowledge-based information on base treatment, preventive actions, and prognoses. In this study, we explore our previous findings of higher sickness absence and disability pension rates for patients with CH further, investigating the occurrence of multimorbidity and whether this is associated with sickness absence and disability pension.

The aim of this study was to explore occurrence of diagnosis-specific multimorbidity among patients with CH and among matched references and possible associations of this with their sickness absence and disability pension.

## Methods

A population-based register study was conducted, using anonymized microdata on patients with CH and matched references. Microdata were obtained from 2 nationwide Swedish registers and linked by Statistics Sweden at the individual level, using the 10-digit personal identification number assigned to all residents in Sweden.^13^

1. The National Board of Health and Welfare's patient registers regarding inpatient and specialized outpatient health care were used to identify individuals who had at least 1 health care visit for CH in 2001–2010 (CH corresponding to code G44.0 in the *International Classification of Diseases, 10th Revision* [ICD-10]^14^). This is a nationwide register and includes data from all private and public organizations that provide secondary health care in Sweden. Inpatient data are based on data available at discharge, including main and secondary diagnoses, as determined by the physician. Regarding the specialist outpatient visits, it is in the same manner the diagnoses arrived at by the treating physician that are included in the register.
2. The Statistics Sweden's Longitudinal Integration Database for Health Insurance and Labour Market Studies (LISA)^15^ was used to identify those patients who in December 2009 were aged 16–64 years and lived in Sweden all of 2010 as well as for identifying the reference group among those with no health care due to CH in 2001–2010. Five individuals were identified for each included patient with CH (N = 16,200) by matching on sex, age (in 5-year categories), type of living area, and educational level, by random sampling. Information about the educational level was missing for 20 (0.6%) of the patients with CH, and they were categorized as having had 0–9 years of schooling. For patients with CH and references, sociodemographics for December 2009 and information on sickness absence and disability pension net days with benefits from the Social Insurance Agency in 2010 were obtained from LISA.

All residents in Sweden aged 16 years and older with income from work or unemployment benefits, whose work capacity is reduced because of morbidity, are covered by the public sickness absence insurance providing benefits. The first sickness absence day is a qualifying day without compensation. For employed, the employer pays for the following 13 days, thereafter, the Swedish Social Insurance Agency pays.^16^ Unemployed can claim such benefits from day 2 of a sickness absence spell. People aged 19–64 years can claim disability pension if their work capacity is long-term or permanently reduced because of morbidity. Both sickness absence and disability pension can be granted for full-time or part-time (100%, 75%, 50%, or 25% of ordinary work hours). That means that people can be on part-time sickness absence and part-time disability pension at the same time, up to 100% of full-time. For the purpose of this study, we used net days in our comparisons, for example, 2 days of absence for 50% of full-time were combined to 1 net day. In this way, we could compare all individuals with sickness absence and/or disability pension, independently of the extent of the respective absences.

Information on multimorbidity was obtained for both patients with CH and references from the inpatient and specialist outpatient health care registers regarding main diagnoses in 2001–2010. Categorizations of diagnoses are summarized in Table 1. Codes R00-R99 and Z00-99 (“Symptoms, signs and abnormal clinical and laboratory findings, not elsewhere classified” and “Factors influencing health status and contact with health services”), except for ICD-10 R25–27, R29, R42, R51, R55–56, and Z03, were excluded from this analysis because of the uncertainty of the diagnosis giving rise to these codes. In some of the analyses, several of the diagnostic groups in Table 1 were collapsed into larger groups.

**Table 1:**
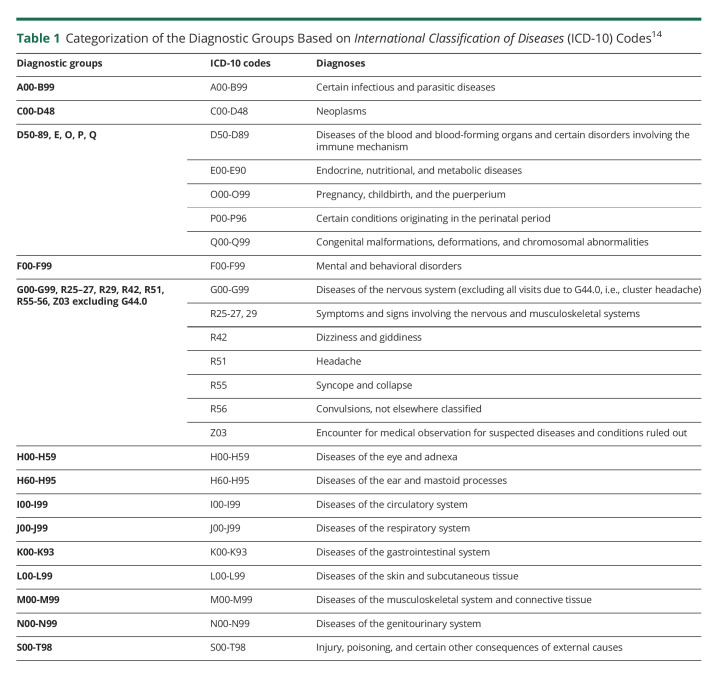
Categorization of the Diagnostic Groups Based on *International Classification of Diseases* (ICD-10) Codes^14^

Multimorbidity among patients with CH was defined as having received secondary health care with at least 1 main diagnostic group in Table 1, other than CH (G44.0). Among references, multimorbidity was defined as having had secondary health care due to at least 2 of the diagnostic categories in Table 1. The analysis concerning sickness absence and disability pension days among individuals in a specific diagnostic group did not exclude individuals from inclusion in other diagnostic groups.

Our outcome measures were (1) proportions of people with multimorbidity among patients with CH and among matched references and odds ratios (OR) computed for group comparisons between patients and references with said morbidities. (2) Prevalence of sickness absence and disability pension and such mean net days in 2010 in CH patients with and without multimorbidity. (3) Proportions of people in 2010 with long-term sickness absence and disability pension benefits, defined as >90 days, >180 days, and >365 days.

### Statistics

Descriptive statistics were used to quantify group differences when applicable. To compare proportions of multimorbidity between groups, we used χ^2^ test, two-tailed *p* values, and a *p* value below 0.05 as a threshold for statistical significance. GraphPad Prism v5.04 was used for analysis (GraphPad Softwares Inc, La Jolla, CA). Logistic regressions were performed in R v.4.0.3 and used to calculate the OR with 95% confidence intervals (CIs) for group comparisons of long-term sickness absence and disability pension.^17^

### Standard Protocol Approvals, Registrations, and Patient Consents

The project was approved by the Swedish Ethical Review Authority and by the authorities administrating the registers, and patient consent was waived by them. In the Nordic countries, patient consents are not applicable in this type of study based on anonymized register data.^18,19^

### Data Availability

Owing to the sensitive nature of the data, they cannot be made public according to Swedish law and legislations (General Data Protection Regulation, the Swedish law SFS 2018:218, the Swedish Data Protection Act, the Swedish Ethical Review Act, and the Public Access to Information and Secrecy Act). The anonymized data used in this study are administered by the Division of Insurance Medicine, Karolinska Institutet. For further inquiries regarding data access, Professor Kristina Alexanderson (kristina.alexanderson@ki.se) may be contacted.

## Results

Detailed descriptive statistics of the cohort comprising 3,240 patients with CH aged 16–64 years and living in Sweden, and 16,200 matched references are reported in Table 2. The majority were men, aged at least 35 years, were born in Sweden, and had at least some high school education.

**Table 2:**
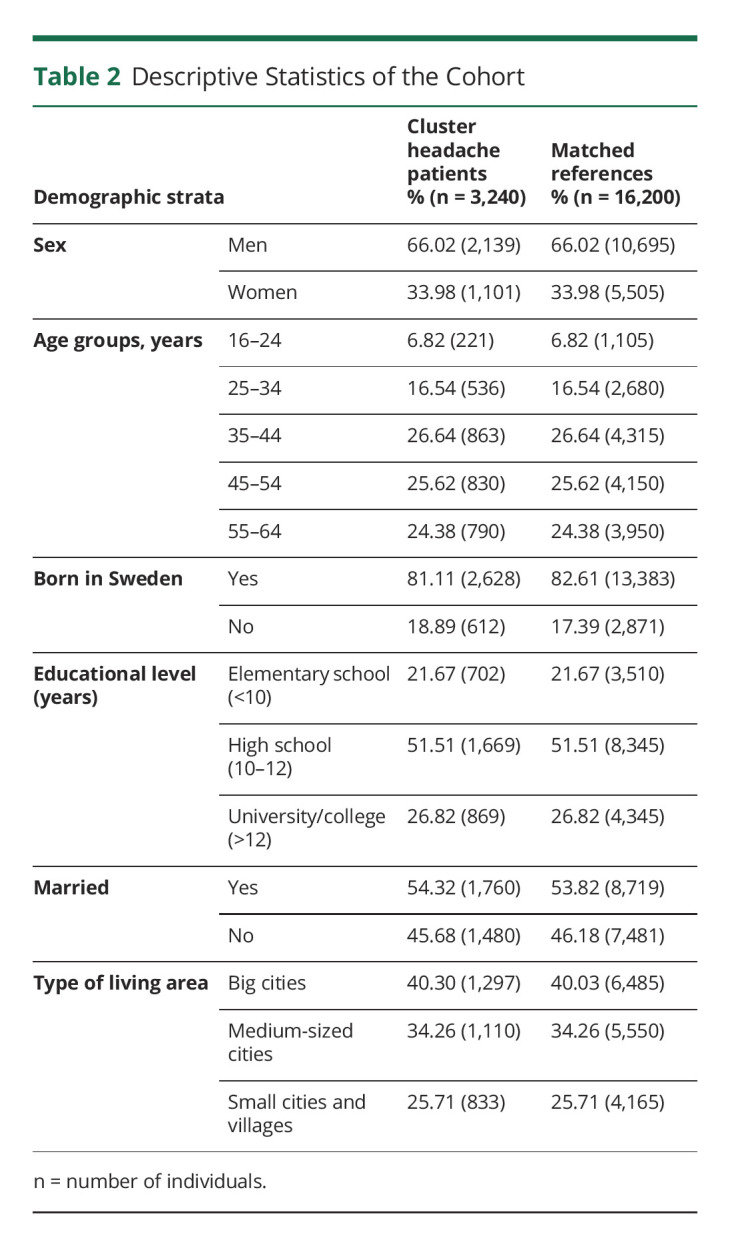
Descriptive Statistics of the Cohort

### Multimorbidity

**Figure 1:**
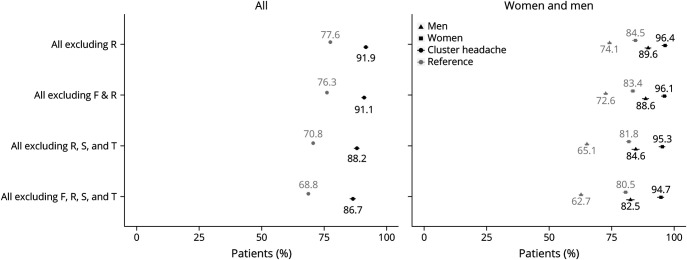
Proportions of Patients With Cluster Headache and Matched References With Multimorbidity Among All and Stratified by Sex, Regarding All Diagnoses and When Excluding Some ICD-10 Diagnostic Categories The occurrence of diagnoses with ICD-10 codes A00-T98 (expressed as percentage of the entire group). Left panel: comparison of individuals with cluster headache (black circles) (n = 3,240) and a matched reference group (gray circles) (n = 16,200). Right panel: stratified analysis of women (black and gray squares) and men (black and gray triangles) with cluster headache and matched references. M: men, W: women, bars: 95% confidence intervals. ICD-10 diagnosis codes; F: mental and behavioral disorders; R: symptoms, signs, and abnormal clinical and laboratory findings, not elsewhere classified, S–T: injury, poisoning, and certain other consequences of external causes.

Among the patients with CH, 91.9% (2,977 patients) had some type of multimorbidity (Figure 1, eTable 1, links.lww.com/WNL/C520). The occurrence of multimorbidity was lower in the reference group, with 77.6% (12,575 individuals) (OR 3.26, 95% CI 2.86–3.72; *p* < 0.0001). If excluding health care for mental and behavioral disorders (ICD-10 codes F00-F99) as a possible source of multimorbidity, 91.1% of the patients with CH still had multimorbidity as compared with 76.3% of the references. When also excluding health care with injuries of external causes (S00-T98), 88.2% of the patients with CH had multimorbidity as compared with 70.8% of the references. Finally, when excluding both these categories of diagnoses (F00-F99 and S00-T98), 86.7% of the patients with CH and 68.8% of references still had multimorbidity, that is, the patients with CH had a higher OR for this than the references (OR 2.95, 95% CI 2.65–3.85; *p* < 0.0001) (Figure 1, eTable 1). Together, these results indicate that a higher proportion of patients with CH had multimorbidity than of the matched references.

Sex-stratified analyses were conducted, revealing that a higher rate of both the women and the men with CH had multimorbidity than that of the matched references (Figure 1). Among patients with CH, a higher proportion of the women had multimorbidity (96.4%) than the men (89.6%). The corresponding proportions among references were 84.5% and 74.1%. However, men with CH seemed to have higher relative load of diagnosis because the ratio (CH vs references) was lower in women (1.14) than in men (1.21). Women remained at particularly high proportions of multimorbidity also when excluding multimorbidity due to mental diagnoses and injury (F00-F99 and S00-T98), with 94.4% in patients with CH as compared with 80.5% in female references (OR 4.35, 95% CI 3.31–5.71, *p* < 0.0001). In men, the corresponding figures were 82.5% in patients and 62.7% in the references (OR 2.81, 95% CI 2.49–3.16, *p* < 0.0001). The relative load of diagnosis was higher in men also after exclusion of the F00-F99 and S00-T98 diagnoses, with a ratio of 1.32 as compared with 1.18 in women.

**Figure 2:**
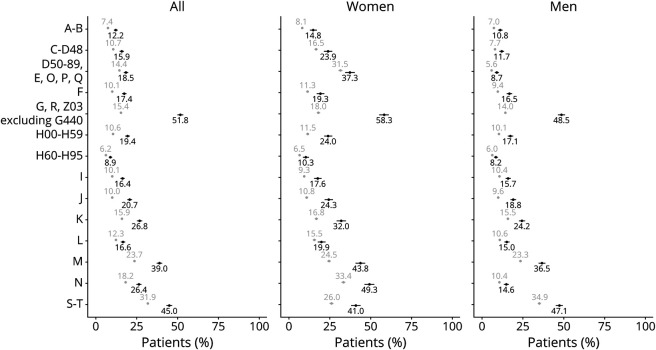
Occurrence of Multimorbidity Among Patients With Cluster Headache and Matched References, Regarding Specific Diagnostic Categories, Among All and Stratified by Sex The occurrence of ICD-10 diagnoses expressed as percentage in a group of individuals with cluster headache (n = 3,240) and a matched reference group (n = 16,200). Left panel: the entire cohort; middle panel: only women; and right panel: only men. Black circles indicate patients with cluster headache, gray circles indicate matched references, and bars: 95% confidence intervals. ICD-10 diagnosis codes; A–B: certain infectious and parasitic diseases; C-D48: neoplasms, D50-D89: diseases of the blood and blood-forming organs and certain disorders involving the immune mechanism; E: endocrine, nutritional, and metabolic diseases; O: pregnancy, childbirth, and the puerperium; P: certain conditions originating in the perinatal period; Q: congenital malformations, deformations, and chromosomal abnormalities; F: mental and behavioral disorders; G: diseases of the nervous system; R: symptoms, signs and abnormal clinical and laboratory findings, not elsewhere classified; Z03: encounter for medical observation for suspected diseases and conditions ruled out; H00-H59: diseases of the eye and adnexa; H60-H95: diseases of the ear and mastoid processes; I: diseases of the circulatory system; J: diseases of the respiratory system; K: diseases of the gastrointestinal system; L: diseases of the skin and subcutaneous tissue; M: diseases of the musculoskeletal system and connective tissue; N: diseases of the genitourinary system; and S–T: injury, poisoning, and certain other consequences of external causes.

When investigating the different groups of ICD-10 diagnoses at a more detailed level, we found that patients with CH consistently had higher rates of multimorbidity with other diagnoses than the reference group (Figure 2, eTable 2, links.lww.com/WNL/C520). Diseases of the nervous system (G00-G99, with G44.0 excluded from analysis) with an OR of 5.9 (95% CI 5.46–6.42) were the diagnostic category with the most pronounced difference. In the CH group, 51.8% had an additional neurologic diagnosis as compared with 15.4% in the reference group. Diagnoses related to diseases of the eye (H00-H59), respiratory (J00-J99), gastrointestinal (K00-K93), and musculoskeletal systems and connective tissue (M00-M99) were also overrepresented in the CH group (Figure 2, eTable 2). In addition, a higher rate of the patients with CH had had secondary health care due to injuries than the references. However, the difference was less pronounced between patients with CH (45.0%) and the references (31.9%) because such injuries were not uncommon in the reference group (OR 1.75, 95% CI 1.62–1.89) (Figure 2, eTable 2). Diseases of the blood and immune system (D50-D89), endocrine and metabolic diseases (E00-E90), and conditions related to pregnancy, childbirth, the perinatal period, and congenital malformations (O00-Q99) were relatively rare and therefore analyzed together. The smallest differences between the 2 groups were found for diseases of the ear and mastoid process (H60-H95) and diseases related to the skin (L00-L99) (Figure 2, eTable 2).

As expected, a higher proportion of the women had had secondary health care with the diagnostic group of ICD-10 codes D, E, O, P, and Q, which includes pregnancy, childbirth, and the puerperium (Figure 2). However, the relative proportions were equal when comparing all patients with CH and references and when comparing the men in these 2 groups. Further sex-stratified analyses revealed a rather big difference in the occurrence of diseases of the genitourinary system (N00-N99) in women; 49.3% in patients with CH compared with 33.4% in the reference group. In addition, diseases of the musculoskeletal systems and connective tissue (M00-M99) were more pronounced among women with CH (43.8%) than among men with CH (36.5%), however, more equal among references (24.5% in women, 23.3% in men). Actually, for all analyzed diagnostic groups, the proportions were higher among women than men, with one exception: multimorbidity including the category external causes of injury (S00-T98) was more common among men (47.1% among men, 41.0% among women with CH; 34.9% among men, 26.0% among women in references).

### Sickness Absence and Disability Pension

The mean number of sickness absence and disability pension net days in 2010 was nearly twice as high among patients with CH than among the references (63.15 [95% CI 58.84–67.45) vs 34.08 days (95% CI 32.59–35.57]). Most of these were days with disability pension in both groups. In addition, the mean net number of days was higher among women in both groups (Table 3). In addition to having more such absence days in all groups included in our comparative analyses, patients with CH had a higher relative proportion of sickness absence days compared with disability pension days (as compared with references) in all groups, except for individuals aged 55–64 years where the number of sickness absence days was relatively low.

**Table 3:**
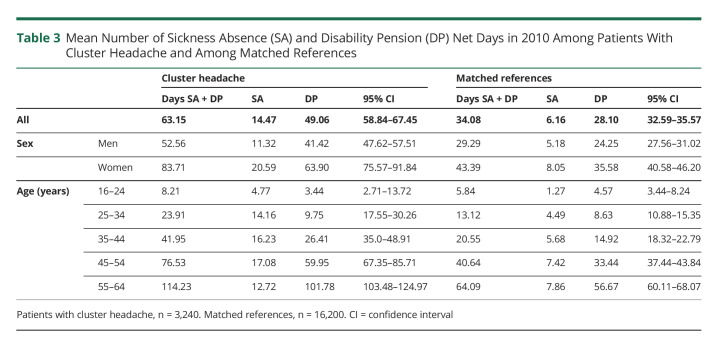
Mean Number of Sickness Absence (SA) and Disability Pension (DP) Net Days in 2010 Among Patients With Cluster Headache and Among Matched References

### Associations of Multimorbidity With Sickness Absence and Disability Pension

For individuals with CH and an additional diagnosis in categories A00-Q99 and S00-T98, the mean number of net days with sickness absence and/or disability pension was much higher than in the CH group without any additional diagnosis, 67.25 days as compared with 16.69 days. The corresponding mean in the reference groups was 40.69 net days in individuals with diagnosis A00-Q99, S00-T98, and 11.16 net days in individuals without any diagnoses (Table 4). Exclusion of diagnoses relating to mental and behavioral disorders and external injuries (F00-F99 and S00-T98) did not significantly change the mean number of net days with sickness absence and/or disability pension in individuals with other diagnoses (69.20 in CH and 40.24 in references). In individuals without other diagnoses, exclusion of diagnoses F00-F99 and S00-T98 resulted in somewhat higher number of days with sickness absence and/or disability pension (23.69 in CH and 16.2 in the reference group) (Table 4).

**Table 4:**
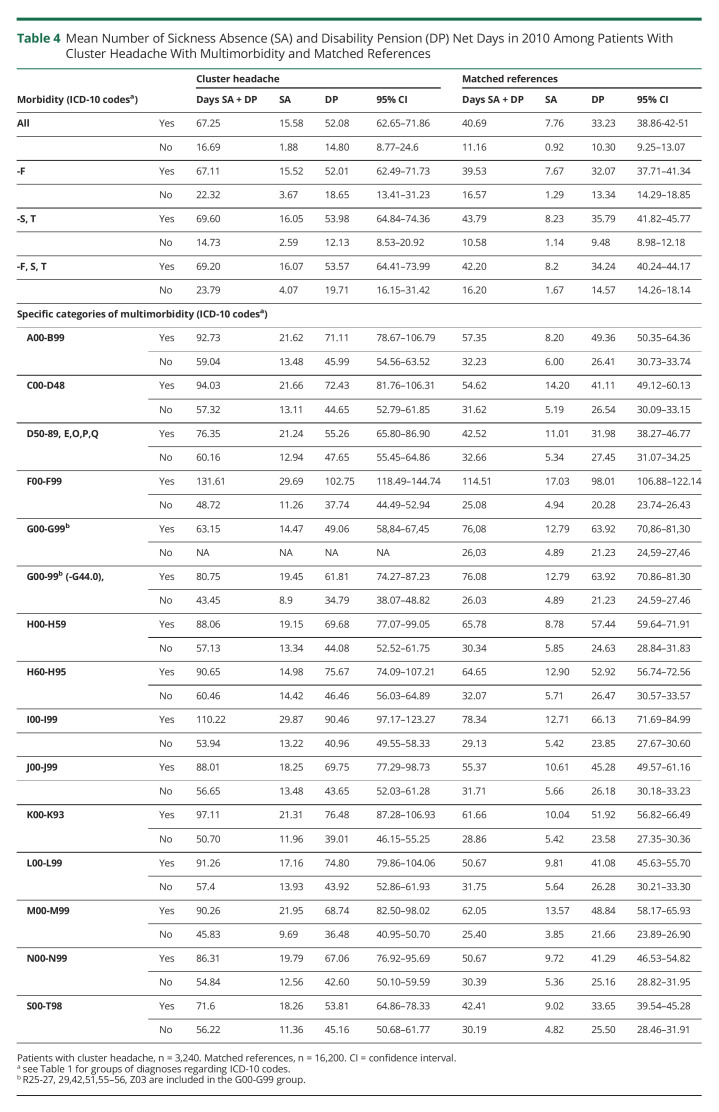
Mean Number of Sickness Absence (SA) and Disability Pension (DP) Net Days in 2010 Among Patients With Cluster Headache With Multimorbidity and Matched References

Detailed investigation of specific multimorbidity and associations with the sickness absence and disability pension of patients with CH showed that CH patients with specific diagnoses were consistently more absent from work as compared with the reference group (Table 4). The difference in mean net days with sickness absence or disability pension was particularly small between the CH patient and reference groups with multimorbidity relating to the nervous system (excluding G44.0, R25–27, R29, R42, R51, R55–56, and Z03 codes) with a mean of 80.75 net days in the patient group vs 76.08 net days for the reference group. Mental and behavioral diagnosis was the group having the highest number of days of sickness absence and disability pension, 131.61 days as compared with 48.72 days without OR = 17.44, 95% CI 14.04–21.67, but the numbers were high also in the reference group, 114.51 days compared with 25.08 days. In addition, CH patients with cardiovascular diseases had high numbers of such absence days, 110.22 days compared with 53.94 days, OR = 13.76, 95% CI 11.03–17.18.

**Figure 3:**
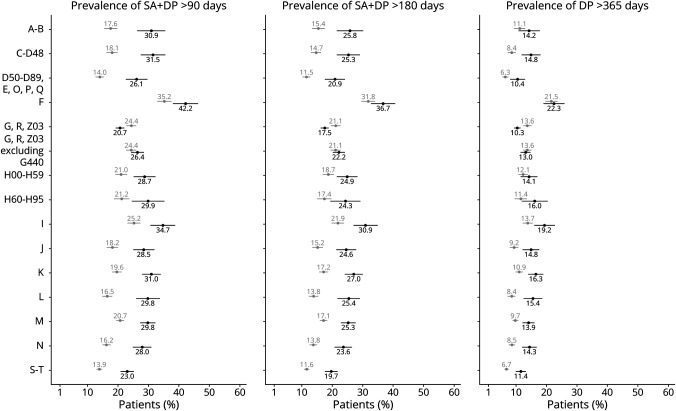
Prevalence of Long-term Sickness Absence and Disability Pension Among Patients With Cluster Headache and Matched References, Respectively, With Different Types of Multimorbidities The prevalence of having had long-term sickness absence (SA) and disability pension (DP) in 2010 in groups with different multimorbidities for more than 90 days (left panel), for more than 180 days (middle panel), and for more than a full year (right panel). Black circles indicate patients with cluster headache (n = 3,240), gray circles indicate matched references (n = 16,200), and bars: 95% confidence intervals. ICD-10 diagnosis codes; A–B: certain infectious and parasitic diseases; C-D48: neoplasms; D50-D89: diseases of the blood and blood-forming organs and certain disorders involving the immune mechanism; E: endocrine, nutritional, and metabolic diseases; O: pregnancy, childbirth, and the puerperium; P: certain conditions originating in the perinatal period; Q: congenital malformations, deformations, and chromosomal abnormalities; F: mental and behavioral disorders; G: diseases of the nervous system; R: symptoms, signs and abnormal clinical and laboratory findings, not elsewhere classified; Z03: encounter for medical observation for suspected diseases and conditions ruled out; H00-H59: diseases of the eye and adnexa; H60-H95: diseases of the ear and mastoid processes; I: diseases of the circulatory system; J: diseases of the respiratory system; K: diseases of the gastrointestinal system; L: diseases of the skin and subcutaneous tissue; M: diseases of the musculoskeletal system and connective tissue; N: diseases of the genitourinary system; and S–T: injury, poisoning, and certain other consequences of external causes.

When exploring the occurrence of having different numbers of sickness absence and/or disability pension days covered by the Swedish Social Insurance Agency in 2010 (>14 days, >90 days, or >180 days) among the patients with CH and among the matched references (Figure 3, eTable 3, links.lww.com/WNL/C520), the prevalence of having any such days of sickness absence and/or disability pension was 30.22% in the CH group as compared with 17.65% in the reference group. In addition, sickness absence and disability pension for extended periods were more frequent in the CH group, with a prevalence of 20.68% for >90 days and 17.5% for >180 days of such absence. This can be compared with 11.06% having >90 days and 9.35% having >180 days in the reference group.

The prevalence of sickness absence and/or disability pension was higher in CH patients with other diagnoses (Figure 3, eTable 4, links.lww.com/WNL/C520). For any number of days, the prevalence of any sickness absence or disability pension was 32.01% (21.07% in the reference group), for >90 days: 21.93% (13.25% in the reference group), and >180 days: 18.78% (11.17% in the reference group). Regardless of the diagnostic category, patients with CH consistently had a higher prevalence of sickness absence and disability pension than the references with the only exception of diseases of the nervous system, where the differences remained small (Figure 3).

We further analyzed the prevalence of having been on disability pension all of 2010 in relation to the presence of multimorbidity (Figure 3). Higher proportions of the patients with CH were on disability pension all the year, with a prevalence of 10.28% in the CH group as compared with 5.82% in the reference group. In CH patients with multimorbidity, this prevalence was 10.98%, while only 2.28% of patients without multimorbidity were on disability pension all 2010.

In-depth investigation of different categories of diagnoses showed that the difference between patients with multimorbidity and references with corresponding diagnoses was less pronounced for >365 days of disability pension than the difference between these groups at >180 days of sickness absence and disability pension (Figure 3).

Mental and behavioral diagnosis (F00-99) was the category most associated with being disability pensioned all the year of 2010; 22.34% in CH patients with an ICD-10 F diagnosis compared with 7.74% in CH patients without. However, the prevalence of being on disability pension the whole year was high also in the reference group with F00-99 multimorbidity (21.47%). Diseases of the circulatory system (I00-99) were also associated with a high prevalence of disability pension the whole year in patients with CH, 19.25% compared with 8.52% in patients without these diagnoses. The corresponding prevalence in the reference group was 13.69% (Figure 3).

## Discussion

By linking data from 2 Swedish population-based registries, we have conducted a study of the occurrence of multimorbidity in patients with CH and the association of multimorbidity with sickness absence and disability pension. The occurrence of multimorbidity was very high in both patients with CH and references; however, it was higher among patients with CH. The proportions were consistent showing higher rates among patients with CH in every diagnostic category analyzed, among all, and among women and men. It is possible that some of the difference may be attributed to a higher tendency among patients with CH to seek health care because they may already have established health care contacts because of their CH.

Patients with CH have been reported to have depression and/or suicidal thoughts when experiencing periods of intense headaches.^3,4^ Our results confirm a higher occurrence of mental disorders in patients with CH; their proportion of having secondary health care due to mental disorders (F) was higher than in the references. However, mental diagnoses did not constitute a major part of the multimorbidity seen in the CH group. The highest numbers of multimorbidity and the most notable difference in comparison with the reference group concerned disorders of the nervous system. Over 50% of the patients with CH had had specialist health care with an additional diagnosis of the nervous system, which may indicate that more patients with CH have neurologic disorders than what was observed in the general population. As discussed above, part of the difference could be related to patients with CH having established health care contact with neurologists, and other neurologic diagnoses may have been discovered during those health care visits. In line with our findings, higher proportions of certain mental and neurologic disorders (e.g., depression, bipolar disorder, migraine, and cerebrovascular disease) were recently reported in a large Norwegian CH register-based study.^20^ Previous studies indicate that diabetes, gastrointestinal problems (K00-93), and cardiovascular diseases (I00-I99) are less common in the CH patient group.^8-10^ Our study did not include comparison of occurrences of individual diagnoses in the 2 groups (CH and references); nevertheless, our results do not indicate lower proportions of these diagnoses in patients with CH, rather the opposite. Multimorbidity with gastrointestinal diseases was found among the diagnosis with a particularly large difference between CH (28.82%) and references (15.93%).

Other diagnoses overrepresented in the CH group were related to diseases of the eye (H00-H59), respiratory (J00-J99), and musculoskeletal systems and connective tissue (M00-M99). Horner syndrome has previously been reported in connection to CH, which would support the high prevalence of eye-related (H) diagnoses.^21^ It should be noted that CH has proven difficult to diagnose and the diagnostic delay can sometimes be several years. Common misdiagnoses have been migraine, dental problems, and sinusitis.^22,23^ Therefore, we cannot exclude that misdiagnosis may account for some of the high rates in the corresponding ICD-10 codes, specifically regarding G, K, and J.

There have been speculations of higher rates of patients with CH engaging in risk-taking behavior, for example, regarding the use of illegal drugs.^24^ In this study, we present data supporting this idea of more risk-taking behavior; higher proportions of the patients with CH had had health care with external injuries.

The world health organization (WHO) estimates that headache is one of the diagnoses that has the most negative effect the quality of life of patients on a global scale, both economically and socially.^25^ Our results support these findings because we can confirm that patients with CH have more days of sickness absence and disability pension but also a higher prevalence of being on such long-term absence from work than the reference group. Having several diagnoses was highly associated with higher sickness absence and disability pension rates; CH patients with multimorbidity had 4 times as many such absence days as compared with CH patients without an additional diagnosis. Mental or cardiovascular (F or I) multimorbidity had the highest association with the number of absence days. Individuals with mental diagnoses had comparably high numbers of absence days in the reference group, indicating that, in Sweden, such diagnoses can have a high effect on the patients' health, work capacity, and life situation, including sickness absence and disability pension. Furthermore, cardiovascular disorders were the second most disabling multimorbidity to CH when comparing the prevalence of long-term sickness absence and disability pension (>180 days) or disability pension only (>365 days), only mental and behavioral disorders were associated with more such days. The difference between CH and matched references regarding cardiovascular diseases is worth noting. We speculate that this may reflect on the difficulties to treat CH in patients with cardiovascular disease. Both verapamil, the first choice of prophylactic medication, and triptans which are efficiently used to abort attacks have cardiovascular contraindications.^26^ Patients with CH more often smoke than the general population,^6,27^ and it is also possible that the high occurrence of smoking in patients with CH negatively affects patients with a combination of CH and cardiovascular disease. Investigating this specific multimorbidity in patients with CH and references who are smokers would be a valuable contribution in future studies.

There are several strengths of our study. First, it was based on microdata obtained from nationwide high-quality registers,^28,29^ linked by unique personal identifiers. This study design implied that all fulfilling the inclusion criteria could be identified, not a sample, that a reference group from the total population, matched on several parameters, could be included, and the large study populations, allowing for subgroup analyses, usually are not possible. Another strength is that all used data were administrative, not self-reported, and affected by, for example, recall bias. A limitation of our study is that we lack information on primary health care events, which may mean an underestimation of some diagnoses and multimorbidity. In addition, personal data susceptible of influencing the occurrence of morbidities, and thereby, our results, for example, smoking, alcohol consumption, or BMI, are missing in our study. Another limitation is that sickness absence days in sickness absence spells ≤14 days were not included. Nevertheless, this was the case for both patients and references. Occurrences of short sickness absence spells might have differed between patients with CH and references, particularly regarding patients who have not yet initiated an effective treatment regime at the onset of an active headache period. Finally, we have chosen to analyze CH under ICD-10 code G44.0, grouping CH patients with episodic CH and CCH. This may be considered a limitation of specificity. In particular for CCH, as patients with CCH are known to have a heavier disease burden, a larger proportion of patients with CCH were reported to receive invalidity allowance in a previous study from Germany.^5^ On the other hand, patients can change phenotype between episodic CH and CCH, and our study design will generate robust results that may be refined by phenotype in future studies. The results regarding sickness absence and disability pension are generalizable to Sweden and other welfare states with high employment frequency. The results regarding multimorbidity in this patient group should be generalizable also to other countries, however, need to be studied also there.

A higher proportion of patients with CH of working age had multimorbidity than of the references in this nationwide study. This was consistent in all categories of diagnostic categories used and was to a higher extent associated with having more sickness absence and disability pension days in patients with CH. Furthermore, the prevalence of long-term sickness absence and disability pension was higher in the CH patient group than in the reference group and highest among those with multimorbidity. It is important that having CH and a cardiovascular disorder stands out as a more disabling combination than other multimorbidities. This study provides very clear indications that CH negatively affects the work capacity of patients and particularly in women and in those having other diagnoses in addition to their CH.

## Glossary

CCH: chronic cluster headache
CH: cluster headache
ICD-10: *International Classification of Diseases, 10th Revision*
